# The antiapoptotic gene survivin is highly expressed in human chondrosarcoma and promotes drug resistance in chondrosarcoma cells *in vitro*

**DOI:** 10.1186/1471-2407-11-120

**Published:** 2011-04-02

**Authors:** Philipp Lechler, Tobias Renkawitz, Valentina Campean, Sanjeevi Balakrishnan, Markus Tingart, Joachim Grifka, Jens Schaumburger

**Affiliations:** 1 Department of Orthopedic Surgery, Regensburg University Medical Center, Asklepios Klinikum Bad Abbach, Bad Abbach, Germany; 2 Department of Pathology, University Hospital of Erlangen, University of Erlangen-Nuremberg, Erlangen, Germany; 3 Department of Internal Medicine, Imperial College London, Hammersmith Campus, London, UK; 4 Department of Orthopedics, RWTH University Hospital, Aachen, Germany

## Abstract

**Background:**

Chondrosarcoma is virtually resistant to chemotherapy and radiation therapy. Survivin, the smallest member of the inhibitor of apoptosis protein family, is a critical factor for tumor progression and resistance to conventional therapeutic approaches in a wide range of malignancies. However, the role of survivin in chondrosarcoma has not been well studied. We examined the importance of survivin gene expression in chondrosarcoma and analysed its influences on proliferation, apoptosis and resistance to chemotherapy *in vitro*.

**Methods:**

Resected chondrosarcoma specimens from which paraffin-embedded tissues could be extracted were available from 12 patients. *In vitro *experiments were performed in human chondrosarcoma cell lines SW1353 and Hs819.T. Immunohistochemistry, immunoblot, quantitative PCR, RNA interference, gene-overexpression and analyses of cell proliferation and apoptosis were performed.

**Results:**

Expression of survivin protein was detected in all chondrosarcoma specimens analyzed, while undetectable in adult human cartilage. RNA interference targeting survivin resulted in a G_2_/M-arrest of the cell cycle and led to increased rates of apoptosis in chondrosarcoma cells *in vitro*. Overexpression of survivin resulted in pronounced resistance to doxorubicin treatment.

**Conclusions:**

These findings indicate that survivin plays a role in the pathogenesis and pronounced chemoresistance of high grade chondrosarcoma. Survivin antagonizing therapeutic strategies may lead to new treatment options in unresectable and metastasized chondrosarcoma.

## Background

Chondrosarcomas comprise a heterogeneous group of neoplasms characterized by the production of cartilage matrix by malignant cells [1] and represent the third most common primary malignancy of bone after myeloma and osteosarcoma [2]. Curative treatment of chondrosarcoma is restricted to surgical resection because of pronounced resistance to chemotherapy and radiation therapy [3-5]. The histological grade is directly related to metastatic rate and remains currently the single relevant predictor of patient outcome [6,7]. After adequate resection, 10-year survival for patients with grade I chondrosarcoma is excellent, whereas only 64% for grade II and 29% for grade III tumors [8]. A large body of evidence has demonstrated that chondrosarcoma's malignant phenotype and resistance to drug therapy is favoured by constitutive activation of antiapoptotic pathways and loss of cell cycle control [9,10].

Survivin, the smallest member of the inhibitor of apoptosis protein (IAP) family is reported to fulfil important roles in cancer initiation, tumor progression and drug/radiation resistance [11]. The molecular structure of survivin reveals one N-terminal baculovirus IAP repeat (BIR) domain and a long C-terminal helix-coiled region. In solution, survivin forms stable homodimers [12,13]. Extensive alternative splicing and a finely, by transcriptional and post-transcriptional mechanisms, controlled expression regulate survivin [14]. It is a bifunctional protein that acts as a suppressor of cell death and plays a key role in cell division. As a chromosomal passenger protein survivin accumulates to kinetochores at metaphase, localizes to the spindle mid-zone at anaphase and is expressed in mid-bodies at telophase [15,16]. While survivin is highly expressed in cancer and during embryonal development it is said to be absent in most adult differentiated organs. Thus, survivin appears to be an ideal therapeutic target for cancer treatment with little toxicity to normal tissues [17]. However, little knowledge exists about expression of survivin in chondrosarcoma [18]. Here, we demonstrate, that the antiapoptotic protein survivin is highly expressed in human high grade chondrosarcoma and possibly acting as a major factor for the tumor's pronounced drug resistance.

## Methods

Unless otherwise stated all chemicals were purchased from Sigma-Aldrich (Taufkirchen, Germany).

The study was approved by the Local Ethics Committee from the University of Regensburg (N° 09-131 and N° 10-101-0170).

### Collection of human tissues

Human chondrosarcoma tissues were collected from radical tumorextirpation, either fixed in 4% para-formaldehyde (immunohistochemistry) or snap frozen (immunoblot). Tumor specimens were analyzed by 2 independent pathologists. Histopathologic diagnosis and tumor grade were confirmed by a national reference pathologist. Detailed patient information can be found on table 1.

**Table 1:** Clinicopathological details of tumor specimens

**Patient Nr**.	Grade	Location	Origin	Size (diameter in cm)	Sex	Age
1	I	upper extremity	peripheral	5	male	68
2	II	costae	central	6	male	53
3	II	pelvis	central	11	female	38
4	II	upper extremity	central	3	male	81
5	III	lower extremity	central	7	male	45
6	III	lower extremity	Central	5	female	78
7	III	pelvis	peripheral	7	male	65
8	III	upper extremity	central	4	female	52
9	III	upper extremity	unclassified	8	female	23
10	III	costae	central	6	female	78
11	III	costae	peripheral	4	male	65
12	III	scapula	unclassified	6	male	71

Clinicopathological data of the 12 chondrosarcomas regarding grade, location, origin, size, patient sex and age.

Non-arthritic human cartilage of 6 Patients undergoing total knee replacement because of mono- or bicompartmental osteoarthritis was collected. The macroscopically and microscopically healthy chondral layer of the unaffected compartment was harvested and either snap frozen or fixed in 4% paraformaldehyde. The mean donor age was 43 years (34 to 56 years). Written informed consent was obtained from each patient.

### Survivin immunohistochemistry

Survivin immunohistochemistry was performed as previously reported [19]. In short, paraffin-embedded specimens were cut into 4 μm sections, dewaxed, and rehydrated in ethanol. Endogenous peroxidase activity was blocked by incubation with 10% H_2_O_2_/phosphate-buffered saline at room temperature. Immunohistochemical staining was performed according to a commercial protocol based on a streptavidin-biotin-peroxidase reaction (DAKO, Hamburg, Germany). For antigen retrieval, sections were cooked for 20 minutes in citrate buffer (pH 6.0) by using a standardized pressure cooker (Biocare Medical, Walnut Creek, CA). Unspecific signals were blocked by incubation with 5% fat-free milk/phosphate-buffered saline for 1 hour at room temperature. Next, sections were incubated with primary antibodies overnight at 4°C. Thorough washing with tris-buffered saline (50 mmol/L Tris-HCl and 136 mmol/L NaCl, pH 7.4) was followed by incubation with biotinylated secondary antibody for 20 minutes. Subsequent to this the slides were incubated with avidin-horseradish peroxidase and the DAB substrate. All incubations were performed in a humidified chamber. Between incubations, specimens were washed three times in tris-buffered saline. All samples were processed in parallel. Omission of primary antibody resulted in completely negative signal. Hematoxylin solution according to Gill was used to counterstain the slides. A Leica DMRB microscope (Leica, Bensheim, Germany) was used to analyse and photograph the specimens. All specimens were stained with rabbit polyclonal antibody AF886 (R&D Systems, Wiesbaden, Germany) and were confirmed with rabbit polyclonal antibody 500.201 (Novus Biologicals, Littleton, CO) and two mouse monoclonal antibodies (clone 60.11 and clone 32.1, Novus Biologicals). Details of all primary and secondary antibodies used are given in table 2.

**Table 2:** Details of antibodies used

Method	Detected protein	Primary antibody	Concentration (μg/ml)	Secondary antibody	Concentration (μg/ml)
					
IHC	Survivin	pAB AF886 (R&D Systems)	5	Goat anti-rabbit immunoglobulin/biotinylated (DAKO)	4
IHC	Survivin	mAB clone 32.1 (Novus Biologicals)	6	Swine anti-mouse immunoglobulin/biotinylated (DAKO)	4
IHC	Survivin	pAB 500.201 (Novus Biologicals)	4	Goat anti-rabbit immunoglobulin/biotinylated (DAKO)	4
IHC	Survivin	mAB clone 60.11 (Novus Biologicals)	7	Swine anti-mouse immunoglobulin/biotinylated (DAKO)	4
IF	Survivin	pAB AF886 (R&D Systems)	2	Red fluorescent dye-labeled anti-rabbit immunoglobulin (Invitrogen)	1
IF	Survivin	mAB clone 60.11 (Novus Biologicals)	2	Red fluorescent dye-labeled anti-mouse immunoglobulin (Invitrogen)	1
IB	Survivin	pAB AF886 (R&D Systems)	1	Swine anti-rabbit immunoglobulins/HRP-conjugated (DAKO)	0,2
IB	Survivin	mAB clone 60.11 (Novus Biologicals)	2	Swine anti-mouse immunoglobulin/HRP-conjugated (DAKO)	0,2

Primary and secondary antibodies used for immunohistochemistry, immunofluorescence, and immunoblotting are listed, with inclusion of sources, purpose, and antibody concentrations. IB - immunoblot, IHC - immunohistochemistry, IF - immunofluorescence;

### Cell line and culture conditions

For cell culture studies the human chondrosarcoma cell lines SW1353 (also known as HTB-94) and Hs 819.T (both obtained from American Type Culture Collection) were cultured in Dulbecco's Modified Eagle Medium (PAA, Cölbe, Germany), supplemented with 10% fetal calf serum, penicillin (50 U/ml) and streptomycin (50 μg/ml). Cells were incubated at 37°C at 5% CO_2 _in humidified air.

### Survivin immunofluorescence

Chondrosarcoma cells were grown on glass slides and fixed over 10 minutes in 3.7% Formalin/PBS at room temperature. Next, sections were cooked for 20 minutes in citrate buffer (pH 6.0, 10 mmol/l). The sections were blocked with phosphatase-buffered saline and 5% fat-free dried milk for 30 minutes at room temperature. After incubation overnight with primary antibody at 4°C and thorough washing with tris-buffered saline, tissues were incubated with red fluorescent dye-labelled anti-rabbit immunoglobulin (Invitrogen, Karlsruhe, Germany) at 37°C for 1 hour. Finally, the nuclei were stained with 4,6-diamidino-2-phenylindole (6.5 g/ml; Invitrogen) for 10 minutes, and the stained sections were analysed and photographed with a fluorescence microscope (Zeiss, Jena, Germany).

### Protein extraction and immunoblot analysis

Protein extraction of tissues and cells was performed as previously described [20]. In brief, cell pellets and tissues were homogenized into extraction buffer (7 mol/L urea, 10% glycerol, 10 mmol/L Tris-HCl, pH 6.8, 1% sodium dodecyl sulfate, 5 mmol/L dithiothreitol, 0.5 mmol/L phenylmethyl sulfonyl fluoride with 1 mg/L aprotinin, pepstatin, and leupeptin) using a T8 Ultra-Turrax homogenizer (IKA, Staufen, Germany). After quantification, protein samples were run on 14% polyacrylamide gels and transferred to Immobilon P membranes (Millipore, Bedford, MA). Unspecific binding-sides were blocked with PBS and 5% fat-free dried milk for 30 minutes at room temperature. Membranes were probed with either polyclonal antibody AF886 (1:1000, R&D Systems) or monoclonal antibody NB500-238 (clone 60.11, 1:500, Novus Biologicals) and horseradish peroxidase-conjugated secondary antibodies (DAKO). Signals were visualized by chemiluminescence (Pierce, Rockford, IL). Recombinant full-length human survivin served as positive control (R&D Systems).

### Survivin knockdown by siRNA

Knockdown of survivin was performed by the transfection of short interfering RNA (siRNA) as described in [19,21,22]. The transfection of human survivin mRNA-specific RNA oligonucleotides suppressed survivin expression effectively at a concentration of 100 nmol/L (suvivin 1). Knock down experiments were confirmed by the application of a second independent pair of siRNA (survivin 2) which resulted in similar reductions of survivin mRNA and protein levels. For negative controls, siRNA targeting green fluorescence protein (GFP) was transfected. 24 hours after knockdown cell cycle distribution and apoptosis were analysed. Sequencences of siRNAs used are given in Table 3.

**Table 3:** Details of siRNA used

Gene	Sense and antisense sequences	Reference
		
Survivin 1	5'-CTTGGCCCAGTGTTTCTTCT-3'	[19,21]
	5'-UGGCUCUUUCUCUGUCCAGTT-3'	
		
Survivin 2	5'-GCGCCUGCACCCCGGAGCG-3'	[22]
	5'-CGCUCCGGGGUGCAGGCGC-3'	
		
GFP	5'-GGUGUGCUGUUUGGAGGUCTT-3'	[20]
	5'-GAACUCCAAACAGCACACCTT-3'	

RNA oligonucleotides with 3'-TT overhangs used for specific gene knockdown. Target gene, sense and antisense sequences and references are listed. GFP - green fluorescent protein;

### Overexpression of survivin

Expression plasmid encoding wild type survivin was generously provided by R. Stauber [23]. One day before transfection, cells were plated at a density of ~50% and expression plasmids were transfected into chondrosarcoma cells using a commercially available transfection reagent (FuGENE^® ^HD, Roche Applied Science, Mannheim, Germany). Conditions according to the manufacturer's instructions. Transfection of pcDNA3 (Invitrogen) served as a negative control. The medium was removed and replaced with full growth medium 6 hours after transfection. The cells were further incubated at 37°C and 5% CO_2 _in humidified air. Transfection efficacy was controlled by immunoblot.

### Cell Cycle Analysis

Both adherent and detached chondrosarcoma cells were collected by trypsinization and washed with PBS for 5 minutes by centrifugation at 125 × g. Cells were resuspended in a staining solution containing 1.5 μmol/L propidium iodide and 25 μg/ml RNase A and incubated for 30 minutes in 37°C. The samples (10000 cells) were analyzed by fluorescence-activated cell sorting with a FACSCalibur (BD Biosciences, Heidelberg, Germany).

### Caspase 3/7 Activity Assay

Apoptosis in chondrosarcoma cells *in vitro *was studied by measuring the activity of caspases 3 and 7 using a commercial kit (Caspase-Glo; Promega, Mannheim, Germany). Cells were seeded in 6 well dishes at 1.5 × 10^5 ^per 3.5 cm well, 24 hours before knockdown was performed. For analysis, 24 hours after knock down cells were incubated for 90 minutes in a luciferase substrate mix. Finally supernatant was removed and cells were homogenized in lysate buffer. Buffer was transferred into a 96-well microplate and luminescence activity was measured in a luminometer (Berthold, Bad Wildbad, Germany). Apoptosis was induced by 24 hours exposure to doxorubicin (5 μM). This concentration resembles the peak plasma level in oncologic patients receiving doxorubicin based treatment regimens [24].

### Measurement of cell viability by MTT

The viability of chondrosarcoma cells was measured by methyl thiazolyl tetrazolium (MTT) assay. Cells were plated onto 96-well plates at a density of 5000 cells per well. 6 hours after transfection with specific siRNA or plasmid, the serum-free medium was replaced by complete medium. The transfection was repeated after 48 hours. MTT reagent (5 mg/ml) in 180 μl medium was added at 0, 24, 48, 72 and 96 hours and incubated for 4 hours at 37°C. Next, supernatant was removed and 150 μl dimethyl sulphoxide (DMSO) was added to each well. After the plate was shaken on a rotary platform for 10 min, extinction at wavelength 490 nm was measured.

### Measurement of cell proliferation

Cell proliferation of chondrosarcoma cells (SW1353 and Hs819.T) was measured by analyzing BrdU incorporation into newly synthesized DNA using a commercially available ELISA chemiluminescence assay (Roche Molecular Biochemicals). Cells were plated out in 96-well microtiterplates at a density of 5000 cells per well and incubated for 24 hours prior the knock down of survivin was performed. 24 after the transfection of specific siRNA the cells were pulsed for BrdU incorporation over 4 hours. ELISA was performed according to the manufacturer's instructions. Chemiluminescence values (relative light units) were measured by an automated luminometer (Berthold, Bad Wildbad, Germany).

### RNA extraction and real-time PCR

Survivin mRNA expression was assayed by performing real-time PCR as described in [19]. In short, RNA was extracted by column purification using the RNeasy micro kit (Qiagen, Hilden, Germany) and RNA transcribed into cDNA. Survivin mRNA expression was detected by a set of intron-spanning primer sequences for human survivin (survivin 1) and was verified by the application of an independent primer set (survivin 2). Control was human β-actin. For primer details see table 4. All primers were applied at a concentration of 300 nmol/L and 55°C annealing temperature. A commercial 2× SYBR Green PCR Mix (Eurogentec, Seraing, Belgium) was used according to the manufacturer's instructions. PCR was performed with 50 cycles, taking 2 μl of cDNA into the reaction with an end volume of 25 μl. Values for survivin were related to their controls using the 2^-Δct ^calculation method.

**Table 4:** Details of primers used for RT-PCR

Gene	Forward and reverse primer sequences	Reference
		
Survivin (1)	5'-AGTGAGGGAGGAAGAAGGCA-3'	[24]
	5'-ATTCACTGTGGAAGGCTCTGC-3'	
		
Survivin (2)	5'-CTTGGCCCAGTGTTTCTTCT-3'	[20]
	5'-CCTCCCAAAGTGCTGGTATT-3'	
		
ß-Actin	5'-AGTCCTGTGGCATCCACGAAA-3'	[20]
	5'-GTCATACTCCTGCTTGCTGA-3'	

Primer sets applied for the detection of survivin and β-Actin mRNA.

### Statistics

At least three replicates for each experimental condition were performed, and the presented results were representative of these replicates. All values are presented as means ± SEM. Student's paired t-test was applied to reveal statistical significances. P values less than 0.05 were considered significant. Statistical analyses were performed using SPSS Software for Windows (version 13.0; SPSS, Inc., Chicago, IL).

## Results

### Survivin is expressed in human chondrosarcoma

**Figure 1:**
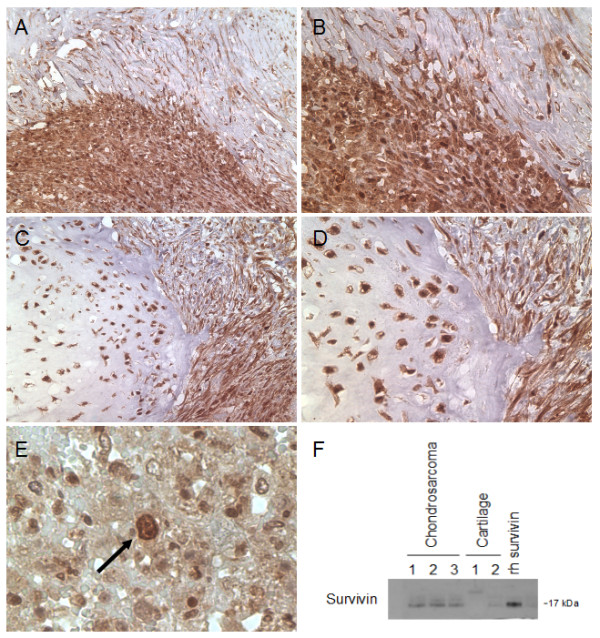
**Survivin expression in human chondrosarcoma**. Immunohistochemistry and immunoblot for survivin (red staining) from human chondrosarcoma specimens. A: Low-power image of human high-grade chondrosarcoma displays strong cellular expression of survivin protein. B: High-power magnification reveals the predominantly cytoplasmic staining, although strong nuclear signals are detectable. C and D: Other specimen of a grade III chondrosarcoma stained with monoclonal antibody, shows a similar pattern of staining. E: Strong survivin signal in a tumor cell displaying a mitotic figure (arrow). F: To verify the expression of survivin in human chondrosarcoma, immunoblots were performed from 3 high grade chondrosarcoma lysates (Patient Nr. 5, 7, 10). As control for the correct molecular weight, in vitro-transcribed and -translated (IVTT) recombinant survivin protein, derived from the full-length human cDNA was loaded. Furthermore, lysates from adult human cartilage served as a negative control. Total protein loaded was 1 μg for recombinant human survivin, 60 μg for chondrosarcoma and cartilage lysates. For A, B and E the polyclonal rabbit anti-survivin antibody AF886 was used. For C and D the monoclonal mouse anti-survivin antibody clone 32.1 was used. Original magnifications: 200× (A and C) and 400× (B and D) and 600× (E).

As a first step, we characterized survivin expression and subcellular distribution in human chondrosarcoma by immunohistochemistry. The staining of paraffin-embedded samples revealed striking expression of survivin protein in all chondrosarcomas analyzed (n = 12) (Figure 1A and 1C). Higher magnification displays the strong, predominantly cytoplasmatic subcellular distribution of survivin protein (Figure 1B and 1D). In grade III chondrosarcoma (n = 8), approximately 30% of visible nuclei stained positive for survivin protein. Importantly, cells displaying mitotic structures and tumor giant cells displayed the strongest staining intensity (Figure 1E). To ascertain the specificity of the pattern of staining, we aimed to verify these findings with several independent antibodies. Altogether, we confirmed the result with two polyclonal (AF866 and pAB 500.201) and two monoclonal (clone 32.1 and clone 60.11) antibodies, where omission of primary antibody gave no signal (data not shown). To strengthen further the evidence of survivin expression in chondrosarcoma we aimed to verify protein expression with techniques other than immunohistochemistry. Hence, tissue lysates of 3 high-grade chondrosarcomas (Patient Nr. 5, 7, 10) showed specific signals for survivin protein by immunoblotting (Figure 1F). To ascertain the correct molecular weight of 16.8 kDa, *in vitro-*transcribed and -translated (IVTT) recombinant survivin protein was loaded. Lysates of adult human articular cartilage (n = 6) served as negative controls. Cartilage number 1 showed a faint band at approximately 28 kDa and cartilage 2 revealed a very weak band at 16.8 kDa. The macro- and microscopically non-arthritic cartilage specimens were obtained from patients undergoing total knee arthroplasty because of mono- or bicompartmental osteoarthritis.

### Survivin is expressed in human chondrosarcoma cells *in vitro *and localizes to heterogenous subcellular compartments

**Figure 2:**
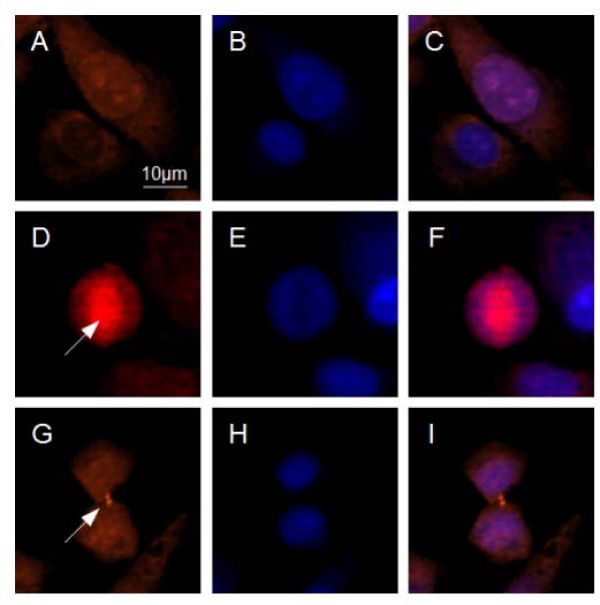
**Survivin subcellular localization in human chondrosarcoma cells *in vitro***. Immunofluorescence localization of survivin using chondrosarcoma cells (SW1353) cultured on glass slides (A,D,G) and 4,6-diamidino-2-phenylindole-staining (DAPI) of the identical positions (B,E,H). Overlay of both stainings (C,F,I). A-C: The top row clearly shows the heterogeneous subcellular distribution from predominant cytoplasmic (lower cell) in the majority of the cell population to mixed cytoplasmic-nuclear in a smaller fraction of cells (upper cell). D-F: In a premitotic cell, survivin localizes to the mitotic spindle apparatus (arrow). Of note, here survivin signal appears stronger compared to the surrounding non-mitotic cells. G-I: In late telophase the mid-body (arrow) stains positive for survivin protein. Original magnifications: 400× (A-I)

Having established that survivin is expressed in human chondrosarcoma, we next examined the survivin expression characteristics in human chondrosarcoma cell line SW1353. Survivin immunofluorescence of SW1353 cells cultured on glass slide revealed a predominantly cytoplasmic localization (~65% of cells) of the protein, while approximately 30% of cells displayed mixed cytoplasmic-nuclear staining (Figure 2A-C). A minor fraction of cells (<5%) showed a predominantly nuclear staining, which may indicate imminent cell division (Figure 2D-F). In less than 1% of cells mitotic structures like spindle apparatus and midbody were seen (Figure 2G-I). Of note, the staining intensity in these cells was by far higher compared to the adjacent, interphasic cells. This finding is consistent with preceding reports describing the mitotic up-regulation of survivin mRNA and protein. Immunofluorescence studies of the human chondrosarcoma cell line Hs 819.T revealed a similar pattern of subcellular survivin protein distribution (data not shown).

### Knock-down of Survivin in chondrosarcoma cells results in reduced rates of proliferation and a failure to exit mitosis

**Figure 3:**
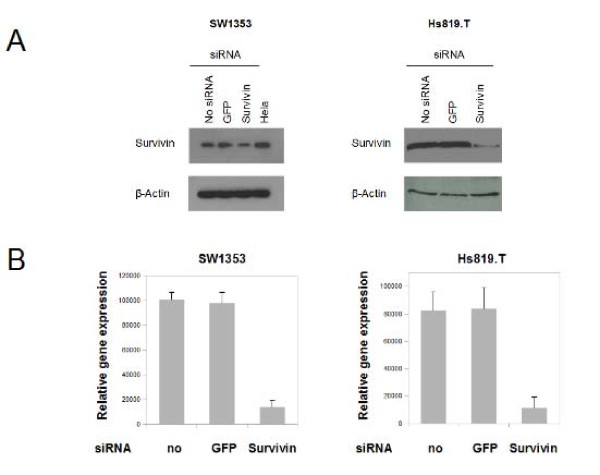
**Suppression of survivin expression by transfection of siRNA**. RNA interference was performed in SW1353 and Hs 819.T, either for GFP as control or for survivin. A: A pronounced decrease of survivin protein levels was measured by immunoblotting in SW1353 and Hs819.T. B: Quantitative real time PCR confirmed the subtotal suppression of survivin expression in SW1353 (left) and Hs819.T (right).

**Figure 4:**
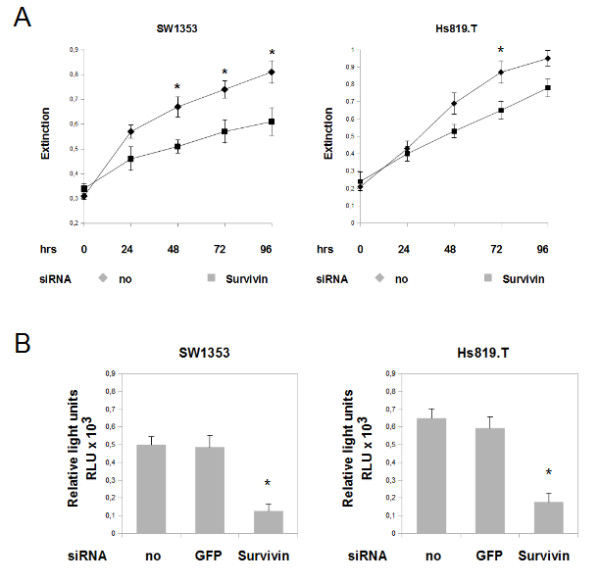
**Influence of survivin knockdown on proliferation and cell viability distribution of chondrosarcoma cells**. The influences of RNA interference with survivin gene expression on cell viability and proliferation were measured by employing the MTT - assay (A) and by measuring BrdU incorporation (B). A: Cell viability was analyzed by MTT assay. Knockdown of survivin was performed at 0 hours and repeated at 48 hours in SW1353 (left) and Hs819.T (right). Significant reduction of viable cells compared to the untransfected control were seen in SW1353 after 48 hours and in Hs819.T at 72 hours.. Knockdown of GFP resulted in no significant alterations (Data not shown). The error bars represent +/-SEM. B: Proliferation of chondrosarcoma cell lines SW1353 and Hs819.T was measured by BrdU incorporation and subsequent detection employing a ELISA chemiluminescence immunoassay. Knock down was performed 24 hours prior to the incubation with BrdU. Knockdown of GFP resulted in no significant alterations. The error bars represent +/-SEM. A: Original results of one representative experiment are shown. B: Original results of three representative experiments are shown. P values less than 0.05 were considered significant (*).

After studying the subcellular localization of survivin protein in chondrosarcoma cell *in vitro*, the functional role of survivin was analysed by using RNA interference. Transfection of survivin specific siRNA resulted in a significant knockdown of survivin protein and mRNA in SW1353 and Hs819.T cells (Figure 3A and 3B). The influence of survivin on cell viability in SW1353 (Figure 4A left) and Hs819.T (Figure 4A right) was analysed by colorimetric measurement of methyl thiazolyl tetrazolium uptake. Knock-down was performed at the beginning of the experiment (0 hours) and repeated on day 2 (48 hours). The MTT-assay revealed a significant lower amount of viable cells 48 hours after the transfection of survivin-specific siRNA (Extinction 0.51, SEM+/-0.03) in SW 1353 compared to the no siRNA control (Extinction 0.64 SEM+/- 0.04). At 72 and 96 hours the reduction of detected viable cells after survivin knockdown was even more pronounced. Transfection of green fluorescent protein (GFP) specific siRNA served as an additional control and lead to no significant alterations of the amount of viable cells (data not shown). Analyzing the effects of survivin knock-down in Hs 819.T revealed a similar tendency towards reduction of measured cell viability (Figure 4A right). To study survivin's influence on cell proliferation in SW 1353 (Figure 3B left) and Hs819.T (Figure 4B right), BrdU incorporation was measured 24 hours after the knock down of survivin. In both cell lines the transfection of survivin specific siRNA led to significantly (p < 0.05) reduced rates of proliferative activity after 24 hours.

**Figure 5:**
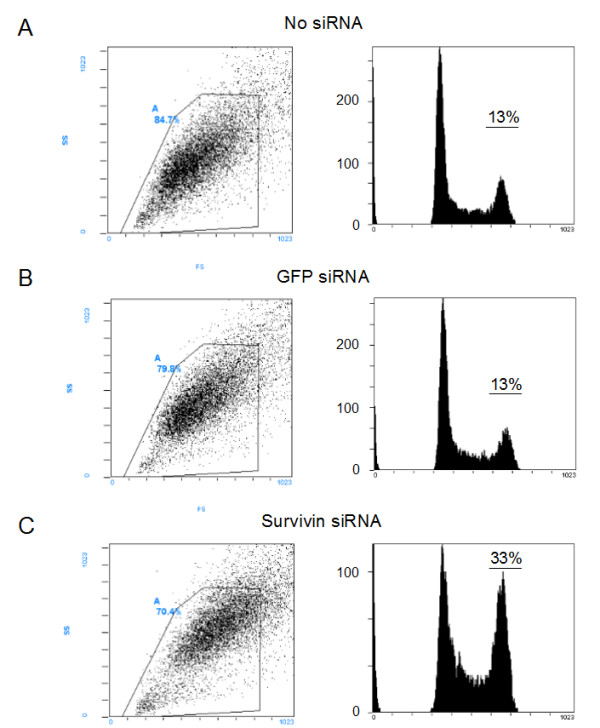
**Effects of survivin knock down on cell cycle distribution in chondrosarcoma cells**. SW1353 were transfected with siRNA targeting survivin and cell cycle distribution was determined by PI staining and FACS analysis after 24 hours. Both attached and detached cells were collected for the FACS analysis. GFP was transfected as control. Original dot blots and measured gates (left) and resulting histograms (right) are shown. The second peak of the resulting histogram represents the G2/M-phase fraction. The original results of one representative experiment are shown.

Cell cycle regulation and involvement in mitotic spindle organization represent well characterized functions of survivin in cancer cells, therefore 24 hours after siRNA transfection in SW1353 cell cultures, cell cycle distribution was analyzed by propidium iodide staining and fluorescence-activated cell sorting (Figure 5). Suppression of survivin resulted in a 2.1 fold increase (SEM+/- 0.3) of the fraction of cells within G_2_/M phase of the cell cycle (Figure 5C). This failure to exit mitosis was previously shown in other tumor cells and underlines survivin's important role in cell division.

### Knockdown of survivin sensitizes chondrosarcoma cells to apoptotic stimuli

**Figure 6:**
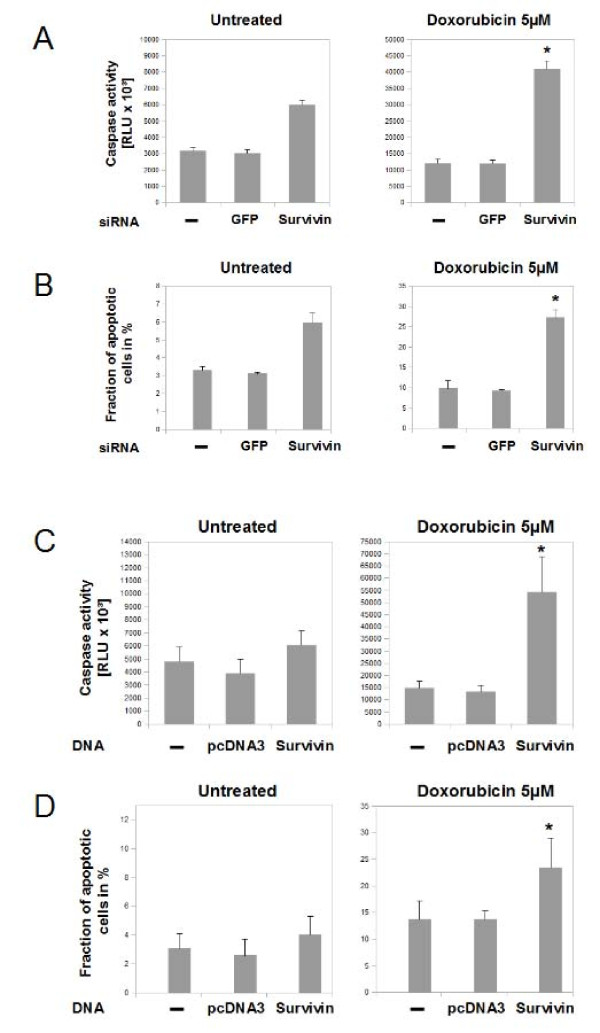
**Influence of survivin knockdown on apoptotic rate of chondrosarcoma cells**. Influences of RNA interference against survivin on programmed cell death of SW1353 (A and B) and Hs819.T (C and D) were measured by caspase 3/7 activity (A and C) and by analysing the sub-G_0/1_-phase fraction by using the fluorescence-activated cell sorting-propidium iodide staining method (B and D). Survivin knockdown resulted in moderate elevations of the indicators of apoptotic activity in SW 1353 (A and B, left) but not in Hs819.T (C and D). Transfection of GFP had no significant effects on apoptosis. Pronounced elevations of apoptotic markers were seen when the cells were stressed with doxorubicin 5 μM over 24 hours (A - D, right). The cytotoxic treatment resulted in a substantial increase of caspase 3/7 activity and fraction of apoptotic cells. Suppression of survivin sensitized the cells to doxorubicin treatment and further increased the apoptotic activity significantly in both cell lines. Again, transfection of GFP siRNA was used as a control. The error bars represent +/-SEM. P values less than 0.05 were considered significant (*). The original results of one representative experiment are shown.

In addition to cell cycle regulation and proliferation, we assayed for influences of survivin on apoptosis by caspase 3/7 activity (Figure 6A and 6C) and propidium iodide staining and fluorescence-activated cell sorting (Figure 6B and 6D). Apoptotic activity was studied 24 hours after survivin knock-down in SW1353 and Hs819.T. Interfering with survivin's function led to an 1.9 fold increase of caspase 3/7 activity and increased the fraction of apoptotic SW 1353 cells 1.8 fold. Next, we tested whether cellular stresses in combination with survivin knockdown revealed a difference. Exposure to 5 μM doxorubicin increased the cellular fraction of apoptotic SW 1353 cells approximately threefold (Figure 6A, right) and caspase 3/7 activity by almost 3.8 fold (Figure 6B, right). Following survivin specific RNA interference in SW 1353 cells doxorubicin exposure (5 μM ) resulted in an 8.3 fold increase of the apoptotic fraction and 12.8 fold increase of caspase 3/7 activity. Next, effects of survivin knock down on apoptosis were analyzed in a second cell line (Hs819.T). While isolated transfection of survivin specific siRNA led to no significant changes in caspase 3/7 activity (Figure 6C, left) or apoptotic fraction (Figure 5D, left), after Doxorubicin exposure the knock down significantly increased both apoptotic markers (Figure 6C and 6D, right).

### Overexpression of survivin protects chondrosarcoma cells against doxorubicin induced apoptosis, but shows no effect on proliferation

**Figure 7:**
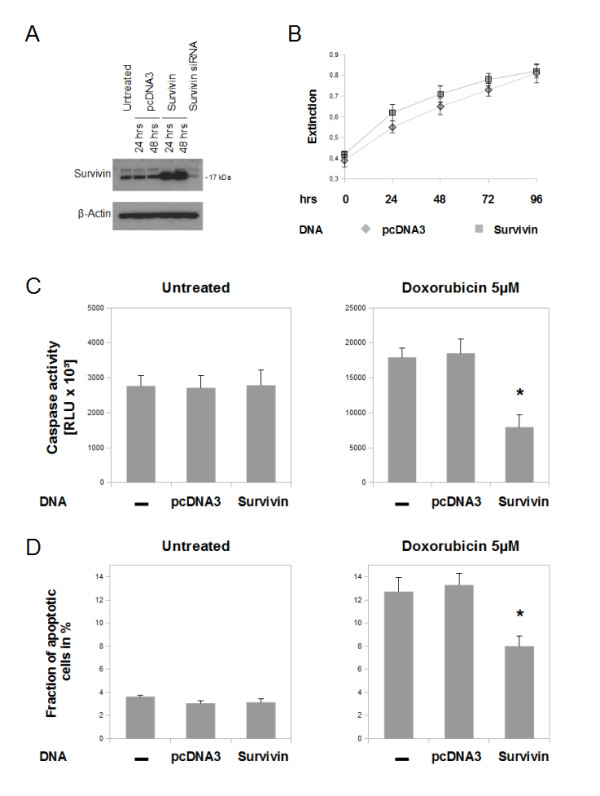
**Influence of survivin overexpression on proliferation and apoptosis of chondrosarcoma cells *in vitro***. A: Overexpression of human full length survivin by transfection of plasmid DNA led to a significant increase of protein level, as measured by immunoblot. Empty vector pcDNA3 was transfected as control. B: MTT - analysis over 5 days after transfection showed no influences on the proliferative activity of SW1353. C and D: Overexpression of survivin resulted in no alterations of spontaneous apoptotic rate as measured by caspase 3/7 activity (C) and by analysing the sub-G_0/1_-phase fraction by using the fluorescence-activated cell sorting-propidium iodide staining method (D). When SW1353 cells were exposed for 24 hours to doxorubicin (5 μM) (right) the apoptotic fraction and caspase activity of not transfected and pcDNA3 transfected cells increased markedly. Transfection of survivin resulted in significantly reduced rates of apoptosis after cytotoxic treatment. The error bars represent +/-SEM. P values less than 0.05 were considered significant (*). The original results of one representative experiment are shown.

Having established that down regulation of survivin gene expression resulted in inhibition of proliferation and increased rates of apoptosis, we next examined the effects of survivin overexpression in SW1353 cells. Overexpression of survivin resulted in a marked upregulation of detectable survivin protein after 24 and 48 hours. While, transfection of empty plasmid (pcDNA3) showed no changes in survivin protein levels (Figure 7A). First, proliferation was analysed by employing the MTT -assay (Figure 7B). Over 96 hours, no significant influences on proliferation were seen at any point of time. Next, we studied the effects of high levels of survivin on apoptosis by caspase 3/7 activity (Figure 7C) and propidium iodide staining and fluorescence-activated cell sorting (Figure 7D). Apoptotic activity was studied 24 hours after transfection with survivin or pcDNA3. Upregulation of survivin led to no significant changes in the spontaneous rate of apoptosis as shown by analysing apoptotic markers (Figure 7C and 7D, left). However, transfection of survivin under cytotoxic conditions (5 μM doxorubicin) reduced both, apoptotic fraction (1.6 fold, Figure 7C, right) and caspase activity (2.2 fold, Figure 7D, right).

## Discussion

Previous studies have shown that survivin, the smallest member of the IAP protein family, has a bifunctional role in cellular division and survival decisions. It is highly expressed at mitosis and is a critical factor for completion of mitotic cell division [15,16]. Survivin acts as a potent inhibitor of apoptotic and non-apoptotic cell death, and protects cells as a stress response factor against unfavourable environments. From a clinical point of view, the most interesting feature of survivin is the widely accepted concept of an "oncofetal" pattern of expression. While undetectable in most adult differentiated tissues, survivin is ubiquitously expressed during embryonal developement and highly re-expressed in cancer. In malignant tumors, survivin antagonizes programmed cell death, favours tumour-associated neovascularization, promotes cell proliferation and preserves cell viability [11]. Disregarding the yet undefined molecular mechanisms, a large body of evidence has demonstrated that survivin has indeed a strong potential of antagonizing drug and radiation induced apoptosis [25,26]. In the current study, we report high expression of survivin in human chondrosarcoma. Furthermore, *in vitro *experiments indicate a potential role in the tumor's pronounced resistance to chemotherapy. Our data shows homogeneous expression of survivin in all analysed human chondrosarcomas (Figure 1A-D), while in adult cartilage no or only low levels of survivin protein were detectable (Figure 1 F). Immunohistochemistry revealed a predominantly cytoplasmic pattern of staining in chondrosarcoma. Immunofluorescence of cultured chondrosarcoma cells confirmed the cytoplasmic subcellular localization of survivin protein (Figure 2), indicating survivin's involvement in extranuclear (i.e. proliferation independent) functions. Of note, recent publications on survivin emphasize the prognostic relevance of subcellular distribution of survivin gene expression. While the prognostic value of nuclear survivin expression in cancer remains unclear, high levels of cytoplasmic survivin protein seem to correlate with resistance to drug/radiation therapy and poor patient outcome [27,28]. The unfavourable prognosis related to cytoplasmic survivin might be associated with its reported extranuclear function (e.g. counteracting apoptosis), whereas nuclear survivin could rather promote cell proliferation [29]. In this context it is of particular interest that effects of strongly active proapoptotic substances as doxorubicin are significantly reduced by survivin overexpression in SW1353 (Figure 7). Accordingly, downregulation of survivin resulted in increased rates of spontaneous and drug induced apoptosis (Figure 6). It is therefore tempting to speculate that survivin represents a key molecule in maintaining constitutive antiapoptotic activity in chondrosarcoma. In this context, it has been shown, that an upregulation of survivin protein did not increase cell proliferation or changed cell cycle distribution, while suppression of survivin resulted in a failure to exit mitosis, the previously described G_2_/M-arrest [21].

## Conclusions

In summary, we demonstrate that the antiapoptotic protein survivin is highly expressed in human high grade chondrosarcoma. Functional analyses in chondrosarcoma cells *in vitro *indicate that survivin exerts the classic functions of cell cycle regulation and survival control in human chondrosarcoma. Moreover, our findings indicate that survivin might be a potent promoter of resistance to chemotherapeutic agents in chondrosarcoma. Still, the role of survivin in oncogenesis and the relevance of its predominantly cytoplasmic distribution in human chondrosarcoma remain elusive.

Learning more about survivin's role in chondrosarcoma and evaluating the effects of survivin-antagonizing therapeutic strategies will be an important task for future studies.

## Abbreviations

IAP: inhibitor of apoptosis protein; BIR: baculovirus inhibitor of apoptosis repeat; MTT: methyl thiazolyl tetrazolium; siRNA: small interfering RNA.

## Competing interests

The authors declare that they have no competing interests.

## Authors' contributions

PL and JS designed research. PL, VC and TR performed experimental study. PL and JS performed statistical analysis. JG and MT participated in data interpretation. PL, JS and JG drafted the manuscript. All authors read and approved the final manuscript.

## Pre-publication history

The pre-publication history for this paper can be accessed here:

http://www.biomedcentral.com/1471-2407/11/120/prepub
